# miR-150-5p and XIST interaction controls monocyte adherence: Implications for osteoarthritis therapy

**DOI:** 10.3389/fimmu.2022.1004334

**Published:** 2022-09-20

**Authors:** Yu-Han Wang, Chun-Hao Tsai, Shan-Chi Liu, Hsien-Te Chen, Jun-Way Chang, Chih-Yuan Ko, Chin-Jung Hsu, Ting-Kuo Chang, Chih-Hsin Tang

**Affiliations:** ^1^ Graduate Institute of Biomedical Science, China Medical University, Taichung, Taiwan; ^2^ Department of Sports Medicine, College of Health Care, China Medical University, Taichung, Taiwan; ^3^ Department of Orthopedic Surgery, China Medical University Hospital, Taichung, Taiwan; ^4^ Department of Medical Education and Research, China Medical University Beigang Hospital, Yunlin, Taiwan; ^5^ The Ph.D. Program of Biotechnology and Biomedical Industry, China Medical University, Taichung, Taiwan; ^6^ School of Chinese Medicine, China Medical University, Taichung, Taiwan; ^7^ Department of Medicine, Mackay Medical College, New Taipei, Taiwan; ^8^ Division of Spine Surgery, Department of Orthopedic Surgery, MacKay Memorial Hospital, New Taipei, Taiwan; ^9^ School of Medicine, China Medical University, Taichung, Taiwan; ^10^ Chinese Medicine Research Center, China Medical University, Taichung, Taiwan; ^11^ Department of Biotechnology, College of Health Science, Asia University, Taichung, Taiwan

**Keywords:** osteoarthritis, miR-150-5p, lncRNA XIST, VCAM-1, monocyte adherence

## Abstract

Recent literature highlights the importance of microRNAs (miRNAs) functioning as diagnostic biomarkers and therapeutic agents in osteoarthritis (OA) and regulators of gene expression. In OA pathogenesis, cell adhesion molecules (CAMs), especially vascular cell adhesion protein 1 (VCAM-1), recruit monocyte infiltration to inflamed synovial tissues and thus accelerate OA progression. Up until now, little has been known about the regulatory mechanisms between miRNAs, long non-coding RNAs (lncRNAs) and VCAM-1 during OA progression. The evidence in this article emphasizes that the functional feature of miR-150-5p is an interaction with the lncRNA X-inactive specific transcript (XIST), which regulates VCAM-1-dependent monocyte adherence in OA synovial fibroblasts (OASFs). Levels of VCAM-1, CD11b (a monocyte marker) and XIST expression were higher in human synovial tissue samples and OASFs, while levels of miR-150-5p were lower in human OA synovial tissue compared with non-OA specimens. XIST enhanced VCAM-1-dependent monocyte adherence to OASFs. Upregulation of miR-150-5p inhibited the effects of XIST upon monocyte adherence. Administration of miR-150-5p effectively ameliorated OA severity in anterior cruciate ligament transection (ACLT) rats. The interaction of miR-150-5p and XIST regulated VCAM-1-dependent monocyte adherence and attenuated OA progression. Our findings suggest that miR-150-5p is a promising small-molecule therapeutic strategy for OA.

## Introduction

Osteoarthritis (OA) is a common chronic joint disorder affecting people worldwide. The prevalence of OA is approximately 15% in Taiwan (1). The clinical symptoms of OA include cartilage degradation, subchondral bone remodeling, and synovial inflammation, resulting in joint pain and disability in OA patients (2). The OA symptoms are typically managed with nonsteroidal anti-inflammatory drugs (NSAIDs), but these have harmful side effects with long-term use (3). There is no effective therapeutic drug for OA, since the precise mechanisms involved in the pathogenesis of OA remain largely unknown. The molecular basis of epigenetics is defined as changes in gene expression by regulatory mechanisms other than changes in the DNA sequence (4). Many studies have proposed that epigenetic mechanisms regulating genes expression are particularly important in OA pathogenesis (5) (6)., The introduction of molecularly targeted therapies that regulate gene expression may be a new strategy for the management of OA (4).

The primary role of the synovium is to maintain joint homeostasis (7). Inflamed synovium has been observed in the early and late stages of OA disease (8). Increasing evidence proposes that synovial inflammation is associated with cartilage damage and contributes to OA severity and progression (9, 10). Synovial cells phagocytose cartilage debris from synovial fluid, leading to the initiation of synovial inflammation. The inflamed synovial cells attract monocyte and macrophage infiltration to the joint, amplifying the inflammatory response and increasing cartilage destruction (11). Cell adhesion molecules (CAMs) regulate monocyte adherence and migration in several inflammatory diseases, including atherosclerosis, rheumatoid arthritis, diabetes, and OA (12–15). In particular, the upregulation of vascular cell adhesion molecule type 1 (VCAM-1) attracts monocyte adherence to human osteoarthritis synovial fibroblasts (OASFs) (16, 17). Reducing the levels of VCAM-1 in synovial fluid improves the inflammatory microenvironment in the OA knee (18). Hence, regulating VCAM-1 expression is recommended as an effective strategy for alleviating OA symptoms (19).

Epigenetic modulation of non-coding RNAs (ncRNAs) has a significant role in regulating gene expression (20). MicroRNAs (miRNAs) and long non-coding RNAs (lncRNAs) are the major ncRNA members (21). The interaction of miRNA and lncRNA regulates the progression of OA by affecting cellular apoptosis (22), proliferation (23) and extracellular matrix degradation (24, 25). Previous studies have indicated that miRNAs regulate CAM expression (e.g., intercellular adhesion molecule 1 [ICAM-1] and VCAM-1) by recognizing the 3′-untranslated region (3′-UTR) of mRNA, affecting inflammatory responses (19, 26). Moreover, lncRNAs can bind with miRNAs and consequently suppress the interaction between miRNAs and their target genes (24, 27). For instance, upregulated expression of lncRNA X-inactive specific transcript (XIST) promotes cell apoptosis and extracellular matrix *via* the miR-149-5p/DNA methyltransferase 3A (DNMT3A) axis in OA cartilage (28). Downregulation of XIST in M1 macrophages contributes to the suppression of OA chondrocyte apoptosis (29). While it is recognized that epigenetic regulation plays a crucial role in OA chondrocytes and is related to OA progression, the underlying mechanism of epigenetic regulation of OASFs remains unclear.

This study aimed to determine whether epigenetic regulation interferes with VCAM-1 expression and is involved in monocyte adherence to OASFs. The levels of CAM and lncRNA expression in OA synovial tissues were analyzed by bioinformatics analysis. We also investigated the effect of VCAM-1 and XIST in OASFs. When we screened for a potential miRNA (miR-150-5p) that not only interacts with XIST but also with VCAM, we found that VCAM-1-mediated monocyte adherence is associated with an interaction between XIST and miR-150-5p. We found that miR-150-5p effectively ameliorated the development of OA disease in joints. Our investigation into the role of miR-150-5p in OASFs suggests a new molecular therapeutic strategy for OA.

## Materials and methods

Materials (including details about the plasmid constructs and the VCAM-1 overexpression [OV] plasmid) and methods relating to clinical samples and primary cell cultures (30), total RNA isolation and the quantitative reverse transcription PCR (RT-qPCR) assay (31), the Western blot assay (32–34), transfection and luciferase reporter assays (35), as well as immunohistochemistry (IHC) staining (36, 37), are all in the **Supplementary Material**.

### *In situ* hybridization (ISH) staining

A digoxigenin-labeled miR-150-5p-modified probe was used to execute ISH staining. ISH staining intensity was scored by two independent observers blinded to the histopathologic data. The staining intensities were categorized as score 0 (no staining or <1 dot per cell), score 1 (1–3 dots per cell, visible at 20–40x), score 2 (4–10 dots per cell and no or very few dot clusters, visible at 20–40x), score 3 (>10 dots per cell and <10% positive cells had dot clusters, visible at 20x), score 4 (>10 dots per cell and >10% positive cells had dot clusters, visible at 20x) (38).

### Bioinformatics analysis

The open-source software libraries Encyclopedia of RNA Interactomes (ENCORI, https://starbase.sysu.edu.cn/) and miRWalk 2.0 (http://mirwalk.umm.uni-heidelberg.de) were searched to predict miRNAs that potentially bind with XIST and VCAM-1. Levels of miRNA expression were analyzed through microRNA sequencing (miRNA-seq) from the Gene Expression Omnibus (GEO) database (GSE143514). Levels of expression were analyzed for CAM and the top 20 lncRNAs in the synovial tissue specimens from 10 healthy donors and 10 patients with OA retrieved from the GEO database (GEO: GDS5401). Levels of lncRNA expression were also analyzed in synovial tissue specimens from 5 patients with OA retrieved from the GEO database (GEO: GDS4195).

### Cell adhesion assay

THP-1 cells were prelabeled with BCECF-AM (10 μM) for 1 h in a serum-free RPMI medium and then used to incubate OASFs for 1 h. OASFs were then gently washed with PBS to remove nonadherent cells and the numbers of adherent cells were quantified under fluorescent microscopy. All experimental procedures were conducted according to our previous research (39, 40).

### RNA pull-down assay

For the biotin-labeled miRNA pull-down assays, we designed biotinylated oligonucleotides with lengths of 22–25 bases (biotinylated negative control [Biotin-NC]; biotinylated miR-150-5p wild-type [Biotin-miR-150-5p WT]; and biotinylated miR-150-5p mutant [Biotin-miR-150-5p MUT]) from sequences provided by MDBio, Inc. (Taipei, Taiwan). The Biotin-NC probe design lacks affinity for the RNA of interest and other RNA sequences in the investigated genome. The Biotin-miR-150-5p WT is a miR-150-5p mimic probe containing a seed region (5’-TCTCCC-’3). Three mismatching nucleotides (CCC to ATT) were incorporated into the biotin-miR-150-5p MUT probe. HEK-293 cells were transfected for 48 h with 200 nM of either Bio-miR-150-5p WT, Bio-miR-150-5p MUT, or Bio-NC. Cells were then lysed in SDS lysis buffer and incubated with M280 magnet streptavidin beads for 4–6 h, then the beads were washed with wash buffer. The biotin-coupled RNAs in the complex were isolated by TRIzol reagent and analyzed by RT-qPCR.

### Experimental OA model

Sprague-Dawley rats (8 weeks of age, weighing around 300–350 g) were acquired from the National Laboratory Animal Center in Taiwan and maintained under conditions described in our previous work (41). All animal procedures were approved by the Institutional Animal Care and Use Committee of China Medical University before their execution (approval number: CMUIACUC-2019-279). OA was induced by following the ACLT protocol established by Wang et al. (42) Lateral arthrotomy was performed in a sterile fashion. The left knee ACL fiber was sectioned and the entire medial meniscus was excised by medial parapatellar mini-arthrotomy. Starting on the same day after surgery, the operated groups were administered single intraperitoneal injections of ampicillin (50 mg/kg of body weight) for 5 days. Sham-operated rats (controls) underwent arthrotomy only and were untreated. All rats were allowed to move freely in the cages and their recovery was monitored by veterinarians. Rats were randomized into groups (n=8 per group) of controls, ACLT, ACLT plus NC mimic, or ACLT plus miR-150-5p mimic. The NC mimic and miR-150-5p mimic groups were administered once-weekly intra-articular injections for 6 weeks. All rats were sacrificed in a CO_2_ chamber at 10 weeks post-surgery and knee joints were collected for micro-computed tomography (micro-CT) imaging and immunohistochemistry (IHC) staining.

### Micro-CT imaging

The micro-CT assessment protocol followed that used in our previous study (16). Rat knee joints were extracted and then fixed using 3.7% formaldehyde for micro-CT imaging. Three-dimensional microstructural volumes from micro-CT scans were analyzed using Skyscan software v1.18 (CTAn/Bruker, Cambridge, UK) (43).

### Statistical analysis

All quantified results are derived from at least three experiments and are presented as the mean ± standard deviation (SD). Statistical analyses were performed using GraphPad Prism 8.0 for Mac (GraphPad Software, La Jolla, CA, USA). The differences between the means of experimental groups were analyzed for statistical significance using a *t*-test for *in vitro* analysis. The one-way ANOVA (two-tail) was followed by Bonferroni testing for *in vivo* analyses. The statistical difference was significant if the *p*-value was < 0.05.

## Results

### Positive correlation between VCAM-1 and CD11b in OA synovial tissue

**Figure 1:**
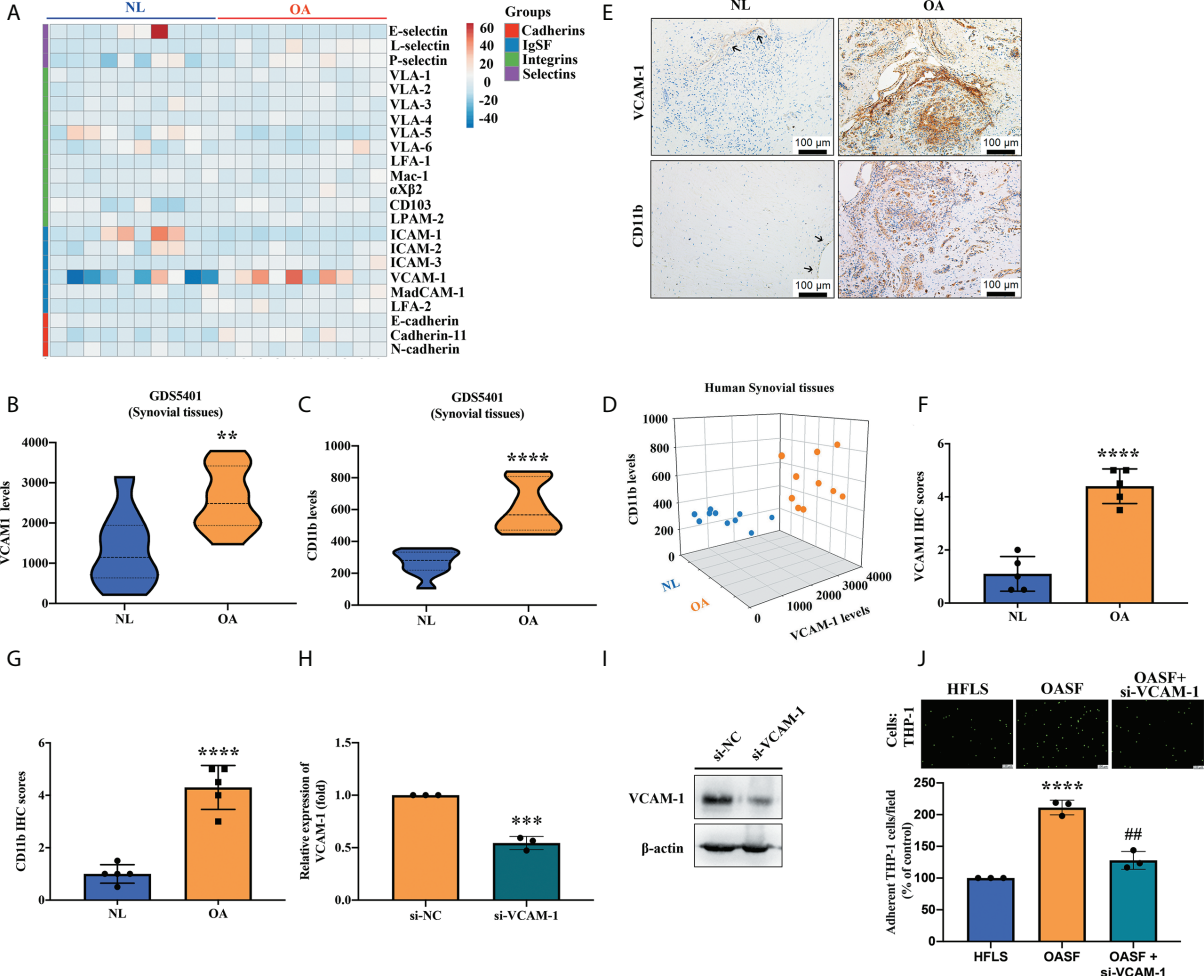
VCAM-1 expression is positively correlated with CD11b expression in OA synovial tissue. **(A)** A cluster heat map generated by a bioinformatics tool used data from the Gene Expression Omnibus (GEO) dataset GDS5401 for analyzing patterns of CAM expression (cadherins, IgSF, integrins, and selectins) in synovial tissue samples (OA patients, n = 10; healthy normal controls, n = 10). **(B, C)** The same dataset was used to analyze expression of VCAM-1 and CD11b in the 10 human OA and 10 normal healthy synovial tissue samples. **(D)** The 2D scatter plot displays the positive relationship between VCAM-1 and CD11b levels in synovial tissue from the 10 OA patients and the 10 healthy normal donors. **(E)** IHC staining was performed to examine levels of VCAM-1 and CD11b expression in hospital samples of OA synovial tissues (n = 5) and healthy synovial tissues (n = 5). Scale bar, 100 μm. **(F, G)** Two independent observers scored the intensity of positive VCAM-1 and CD11b expression on a scale of 1 (weak) to 5 (strong). **(H, I)** OASFs were transfected with VCAM-1 siRNA for 24 h, then VCAM-1 mRNA and protein levels were quantified by RT-qPCR (n = 3) and Western blot (n = 3). **(J)** Monocytes were labeled with green fluorescence (BCECF-AM) for 1 h, then incubated with OASFs for 1 h. Monocytes were photographed under fluorescence microscopy. The images were quantified for adherent cells using ImageJ software in three independent experiments. LFA, lymphocyte function-associated antigen; VLA, very late antigen; Mac-1; macrophage-1 antigen; CD103, cluster of differentiation 103; LPAM-2, lymphocyte Peyer’s patch adhesion molecule 2; ICAM, intercellular adhesion molecule; VCAM; vascular cell adhesion protein; MAdCAM-1; mucosal vascular addressin cell adhesion molecule 1; OASF; osteoarthritis synovial fibroblast; HFLS, human fibroblast-like synoviocyte. ^**^ *p* < 0.01, ^***^ *p* < 0.001 and ^****^ *p* < 0.0001 compared with healthy normal controls; ^##^ *p* < 0.01 compared with OASFs.

To investigate the relevance of CAMs and monocytes in OA patients, we first analyzed gene expression profiles from the GEO database repository. Levels of CAM expression in healthy donor and OA synovial tissues are shown in **Figure 1A**. Levels of VCAM-1 expression were significantly higher than levels of other CAMs (cadherins, integrins and selectins) in OA synovium compared with normal non-OA synovium (**Figure 1B**). When we analyzed the levels of monocyte surface marker CD11b, we found markedly higher levels of CD11b expression in synovial tissue specimens from OA patients than in healthy individuals (**Figure 1C**). Moreover, we found a positive correlation between VCAM-1 expression and levels of CD11b expression in synovial tissue from patients with OA compared with normal synovial tissue (**Figure 1D**). Similarly, our IHC analyses revealed substantially higher VCAM-1 and CD11b expression in synovial tissue from OA patients than in normal healthy synovial tissue (**Figures 1E–G**). To examine the functional role of VCAM-1 in OASFs, we transfected OASFs with the VCAM-1 siRNA for 24 h to determine monocyte adherent ability. Analyses found that the VCAM-1 siRNA reduced VCAM-1 mRNA and protein levels (**Figures 1H, I**). A higher number of THP-1 monocytes compared with HFLSs were adherent to OASFs (**Figure 1J**). VCAM-1 downregulation significantly reduced monocyte adherence to OASFs (**Figure 1J**). We suggest that VCAM-1 may be a critical mediator in the regulation of monocyte adherence to OASFs.

### XIST expression affects VCAM-1-mediated monocyte adherence

**Figure 2:**
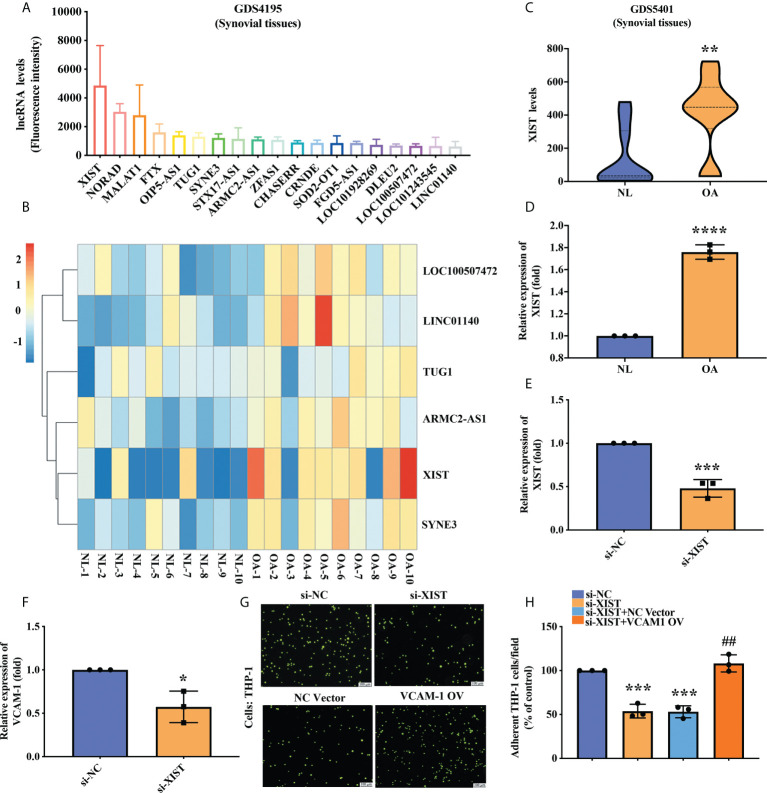
Higher levels of XIST expression in OA synovial tissue and knockdown of XIST inhibited VCAM-1-mediated monocyte adherence. **(A)** An analysis of the GEO dataset GDS4195 identified the top 20 upregulated lncRNAs in human OA synovial tissue specimens (n = 5). **(B)** A cluster heat map shows lncRNA expression in human synovial tissue (red denotes higher expression and blue denotes lower expression) from the GEO dataset GDS5401. **(C)** Levels of XIST expression in OA (n = 10) and healthy synovial tissue (n = 10) samples from GDS5401. **(D)** Total RNA for each sample of human synovium was extracted using TRIzol reagent. Levels of XIST were quantified by RT-qPCR assay (n = 3). **(E, F)** OASFs were transfected with XIST siRNA for 24 h. The RT-qPCR assay measured XIST and VCAM-1 expression (n = 3). **(G)** OASFs were co-transfected with XIST siRNA and the VCAM-1 OV plasmid for 24 h. THP-1 cells were labeled with the green fluorescence BCECF-AM (10 μM) for 1 h. After washing the unlabeled THP-1 cells, they were resuspended in a serum-free RPMI medium and then co-cultured with OASFs for 1 h. Fluorescence intensity was measured by Zeiss AxioPhot fluorescence microscopy in the images and quantified by ImageJ processing software in three independent experiments **(H)**. TUG1, taurine upregulated gene 1. ^*^ *p* < 0.05, ^**^ *p* < 0.01, ^***^ *p* < 0.001, ^****^ *p* < 0.0001 compared with control siRNA; ^##^ *p* < 0.01 compared with XIST siRNA.

lncRNAs have a critical role in gene regulation and their dysregulation is generally associated with disease initiation and development (20). First, we identified the top 20 lncRNAs that were upregulated in human OA synovial tissue specimens downloaded from the GEO dataset GDS4195; the levels of expression for six of those lncRNAs were higher than all the other lncRNAs (**Figure 2A**). These six lncRNAs were also highly expressed in OA synovial tissue compared with healthy tissue samples obtained from the GEO dataset GDS5401 (**Figure 2B**); levels of XIST expression were significantly higher in OA synovial tissue compared with normal tissue (**Figure 2C**). In our tissue samples from hospital patients, RT-qPCR analysis revealed significantly higher levels of XIST expression in OA tissue than in healthy tissue (**Figure 2D**). To investigate whether alterations in XIST expression affect VCAM-1 expression and the ability of monocytes to adhere to OASFs, we transfected OASFs with XIST siRNA for 24 h. We found that XIST siRNA significantly inhibited levels of both XIST and VCAM-1 expression (**Figures 2E, F**). Transfecting OASFs with XIST siRNA downregulated XIST and inhibited the attachment of monocytes to OASFs, but when the VCAM-1 OV plasmid was co-transfected with XIST siRNA, the effects of XIST siRNA were significantly reversed (**Figures 2G, H**). These results suggest that higher levels of XIST in OA synovium enhance VCAM-1 expression and promote monocyte adherence to OASFs.

### Upregulation of miR-150-5p impedes VCAM1-mediated monocyte adherence

**Figure 3:**
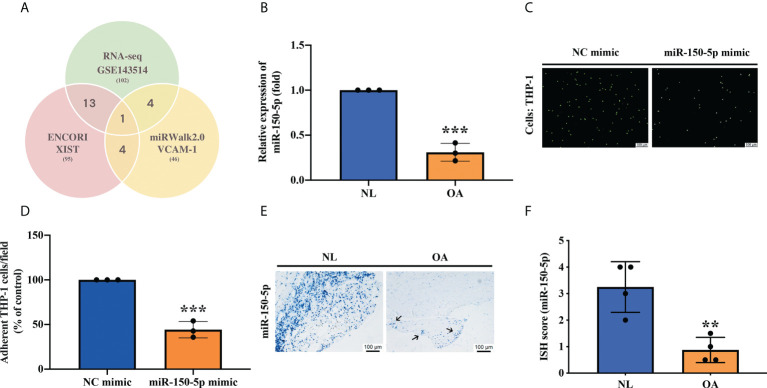
miR-150-5p is downregulated in OA synovial tissue and promotes monocyte adherence. **(A)** The schematic for miRNA filtering: miRNA-seq analysis identified downregulated levels of miRNA expression in OA synovial tissue. Open-source platforms ENCORI and miRWalk2.0 were analyzed to predict miRNAs that target VCAM-1 and XIST. **(B)** RT-qPCR revealed levels of miR-150-5p expression in 3 samples of normal healthy synovial tissue and 3 samples of OA synovial tissue. **(C, D)** OASFs were transfected with miR-150-5p mimic for 24 h. BCECF-AM labeled THP-1 cells (2×10 (4) cells/ml) were incubated with OASFs for 1 h. The images of adherent THP-1 cells were photographed under fluorescence microscopy and then quantified by ImageJ software in three independent experiments. **(E, F)** The blue dots indicate levels of miR-150-5p expression in 4 images of human synovial tissue and quantification by ISH scores, from 0 (no staining or <1 dot per cell) to 4 (>10 dots per cell and >10% positive cells had dot clusters, visible at 20x). ^**^ *p* < 0.01, ^***^ *p* < 0.001 compared with normal synovial tissue or control mimic.

As miRNAs can regulate biological pathways by interacting with several molecules (44), we examined miRNA-seq data in the GEO dataset and open-source software (ENCORI and miRWalk2.0) to predict which miRNAs are involved in XIST and VCAM-1 regulation. First, we filtered miRNAs by the species (human/rat) that corresponded to our experiment. Of a total of 102 miRNAs that were downregulated in OA synovial tissue compared with normal synovial tissue specimens from the GEO dataset, miR-150-5p was the only miRNA to have binding sites in XIST and VCAM-1, both of which showed high affinity for miR-150-5p (**Figure 3A**). Analyses of our human OA and non-OA synovium samples revealed that miR-150-5p expression was markedly reduced in OA synovium compared with non-OA synovium (**Figure 3B**). To further investigate the role of miR-150-5p in OASFs, we transfected OASFs with miR-150-5p mimics. We found that miR-150-5p effectively suppressed monocyte adherence to OASFs (**Figures 3C, D**). In addition, ISH staining demonstrated significantly lower levels of miR-150-5p expression in OA synovial tissue compared with normal synovial tissue (**Figures 3E, F**). These data suggest that miR-150-5p is a crucial (negative) regulator of monocyte adherence to OASFs.

**Figure 4:**
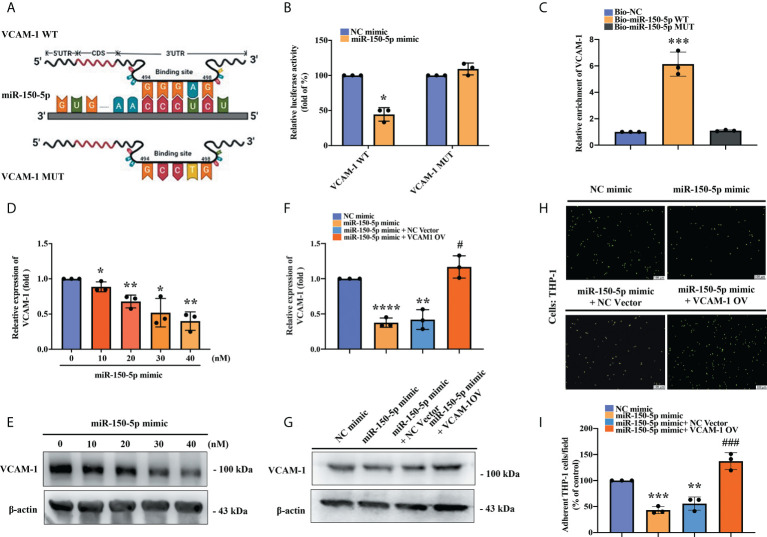
An interaction between miR-150-5p and VCAM-1 mitigates monocyte adherence in OASFs. **(A)** The schematic diagram shows the sequence binding site of miRNA-150-5p with VCAM-1 mRNA in miRWalk2.0. **(B)** The VCAM-1 WT plasmid and VCAM-1 MUT plasmid were each co-transfected with miRNA-150-5p mimic in OASFs for 24 h. Luciferase activities were measured and analyzed using the luminometer reader in three independent experiments. **(C)** After transfecting HEK-293 cells with each biotinylated probe, the lysed cells were incubated with M280 magnetic streptavidin beads (200 nM) for 4–6 (h) RNAs were isolated by TRIzol reagent, and levels of VCAM-1 expression were detected using RT-qPCR in samples pulled down by biotinylated miR-150-5p or negative control in three independent experiments. **(D, E)** OASFs were transfected with miR-150-5p mimic (0, 10, 20, 30, 40, or 50 nmol/L) for 24 h, and the levels of VCAM-1 expression were examined by RT-qPCR and Western blot in three independent experiments. **(F, G)** OASFs were co-transfected with miR-150-5p mimic and the VCAM-1 OV plasmid. VCAM-1 mRNA and protein levels were detected by RT-qPCR and Western blot in three independent experiments. **(H, I)** THP-1 cells were labeled with BCECF-AM and then incubated with OASFs for 1 h. The numbers of adherent cells were qualified by Zeiss AxioPhot fluorescence microscopy in three independent experiments. ^*^ *p* < 0.05, ^**^ *p* < 0.01, ^***^ *p* < 0.001, ^****^ *p* < 0.0001 compared with control mimic; ^#^ *p* < 0.05, ^###^ *p* < 0.001 compared with miR-150-5p mimic.

To verify whether miR-150-5p binding with VCAM-1 3’-UTR affects VCAM-1 transcription, we constructed the VCAM-1 WT andVCAM-1 MUT luciferase reporter plasmids (**Figure 4A**). These were co-transfected into OASFs with miR-150-5p mimic or NC mimic. We found that miR‐150-5p significantly reduced luciferase activity of the VCAM-1 WT reporter, whereas there was no significant difference in VCAM-1 MUT reporter activity after either miR-150-5p mimic or NC mimic transfection (**Figure 4B**). We used a high-efficiency transfection cell line HEK-293 to examine whether miR-150-5p interferes with VCAM-1 transcription in the RNA pull-down assay. The results revealed that in contrast to the Biotin-miR-150-5p MUT probe, the binding of VCAM-1 to the Biotin-miR-150-5p WT probe was significantly enhanced (**Figure 4C**). We then transfected OASFs with miR-150-5p mimic and identified significant, dose‐dependent effects on VCAM-1 mRNA and protein expression in RT-qPCR and Western blot assays (**Figures 4D, E**). We then examined the effects of miR-150-5p mimic on VCAM-1 expression and monocyte adherence to OASFs. Administration of miR-150-5p mimic significantly inhibited VCAM-1 mRNA and protein levels and monocyte adherence to OASFs; all of these effects were reversed when OASFs were transfected with the VCAM-1 OV plasmid (**Figures 4F–I**). These results revealed that miR-150-5p directly suppresses VCAM-1 expression, reducing monocyte adherence to OASFs.

### miR-150-5p downregulation blockaded the effect of downregulated XIST on VCAM-1-derived monocyte adherence

**Figure 5:**
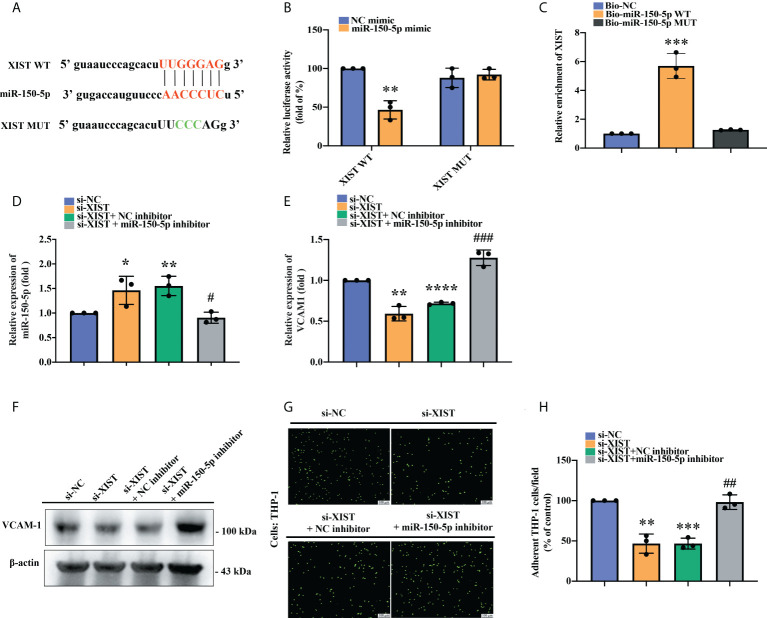
The effect of XIST knockdown on monocyte adherence was blocked by miR-150-5p downregulation. **(A)** The schematic diagram illustrates the sequences of miRNA-150-5p interactions with XIST in the ENCORI database. **(B)** A luciferase reporter assay was performed to confirm the interaction between miR-150-5p and XIST. The XIST WT plasmid and XIST MUT plasmid were each co-transfected with miRNA-150-5p mimic into OASFs for 24 h. Luciferase activities were determined by the luminometer in three independent experiments. **(C)** The RNA pull-down assay revealed direct binding of miR-150-5p with XIST. The biotinylated probes (Biotin-NC, Biotin-miR-150-5p WT, Biotin-miR-150-5p MUT) were transfected into HEK-293 cells for 48 h. The cells were lysed and incubated with M280 magnetic streptavidin beads for 4–6 (h) The biotin-coupled RNAs were isolated using TRIzol reagent and measured by RT-qPCR assay in three independent experiments. **(D)** miR-150-5p expression was examined by RT-qPCR after OASFs were co-transfected with XIST siRNA and the miR-150-5p inhibitor for 24 h in three independent experiments. **(E, F)** After transfecting OASFs with XIST siRNA and the miR-150-5p inhibitor, the cells were subjected to RT-qPCR for mRNA expression and to Western blot for protein expression in three independent experiments. **(G, H)** OASFs were transfected with XIST siRNA, miR-150-5p inhibitor, or their respective controls for 24 h. BCECF-AM labeled THP-1 cells were co-cultured with OASFs for 1 h, then adherent THP-1 cells were determined by fluorescence microscopy in three independent experiments. ^*^ *p* < 0.05 and ^**^ *p* < 0.01, ^***^ *p* < 0.001, ^****^ *p* < 0.0001 compared with control siRNA or control mimic; ^#^ *p* < 0.05, ^##^ *p* < 0.01, ^###^ *p* < 0.001 compared with XIST siRNA.

Next, we examined the interplay between miR-150-5p and XIST. The open-source platform ENCORI was used to predict the XIST and miR-150-5p binding site and a mutant sequence of XIST was designed (**Figure 5A**). The luciferase reporter assay revealed that the luciferase activity of the XIST WT vector was weakened by miR-150-5p upregulation, whereas the activity of the XIST-MUT vector was not influenced (**Figure 5B**). XIST expression was remarkably enhanced in the WT biotinylated miR-150-5p probe compared with the MUT biotinylated miR-150-5p probe in the RNA pull-down assay (**Figure 5C**). These results confirmed the interaction between XIST and miR-150-5p at the predicted site. We further explored the impact of the miR-150-5p and XIST interaction upon VCAM-1-dependent monocyte adherence to OASFs. We observed that miR-150-5p expression was significantly upregulated after OASFs were transfected with XIST siRNA and was inhibited after OASFs were transfected with the miR-150-5p inhibitor (**Figure 5D**). Furthermore, we found that while VCAM-1 mRNA and protein expression was inhibited after XIST expression was downregulated in OASFs, this phenomenon was reversed after the OASFs were transfected with the miR-150-5p inhibitor. Similarly, we found that downregulating XIST suppressed monocyte adherence ability and that this effect was reversed when OASFs were transfected with the miR-150-5p inhibitor (**Figures 5E–H**). These results suggest that miR-150-5p plays an essential role in monocyte adherence to OASFs.

### Upregulation of miR-150-5p ameliorates histological severity of OA

**Figure 6:**
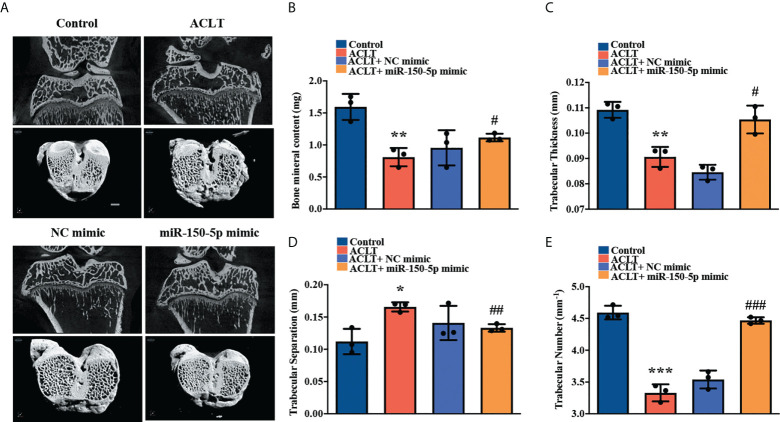
miR-150-5p alleviates the severity of OA in rats. **(A)** Micro‐CT images from subchondral bone in rat knees from each group (Control, ACLT, ACLT + NC mimic, and ACLT + miR-150-5p mimic) were scanned using SkyScan micro-CT scanners. Micro-CT parameters analyzed **(B)** the percentage of subchondral bone volume in tissue volume, **(C)** trabecular thickness, **(D)** trabecular separation, and **(E)** trabecular numbers in all rats, in three independent experiments. ^*^ *p* < 0.05, ^**^ *p* < 0.01, ^***^ *p* < 0.001 compared with Control; ^#^ *p* < 0.05, ^##^ *p* < 0.01, ^###^ *p* < 0.001 compared with ACLT rats.

We established the ACLT model to validate the role of miR-150-5p *in vivo*. The rats were injected with synthetic miR-150-5p mimic (5 nmol) or NC mimic once a week. Six weeks later, all rats were sacrificed, and the knees were collected for micro-CT scanning and tissue staining analysis. Micro-CT images revealed that in contrast to the control group, severe subchondral bone erosion was observed in the ACLT and the ACLT + NC mimic groups; less joint destruction was observed in knees administered miR-150-5p mimic (**Figure 6A**). Quantitative analysis of the micro-CT findings revealed that bone mineral content (BMC), trabecular thickness, and trabecular numbers were significantly decreased, while there were significant increases in trabecular separation in the ACLT and ACLT + NC mimic groups compared with the control group (**Figures 6B–E**). Administration of miR-150-5p mimic markedly reversed ACLT-induced effects (**Figures 6B–E**).

**Figure 7:**
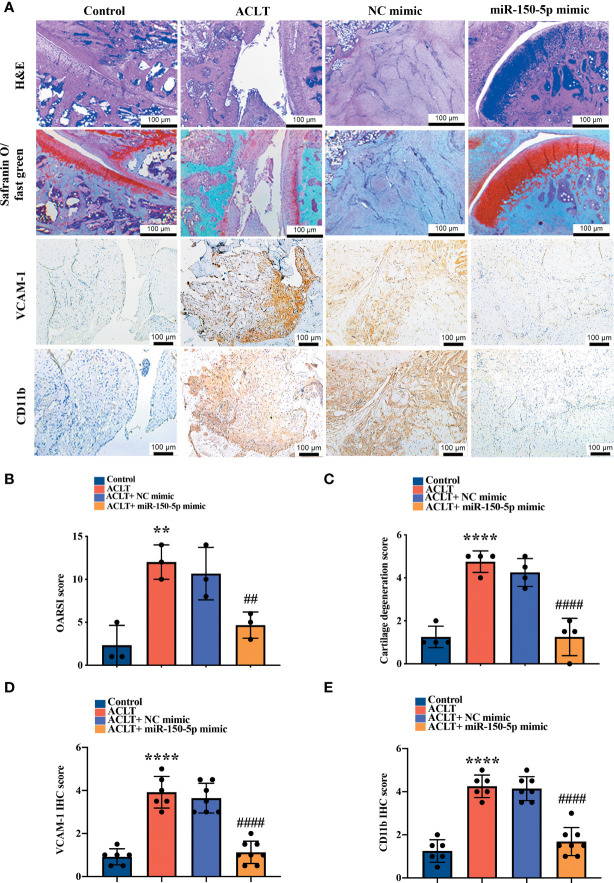
Histopathological evidence reveals that miR-150-5p ameliorates OA progression. **(A)** Specimens from the four groups were immunostained with H&E in four independent experiments, Safranin-O (four independent experiments), VCAM-1 (six independent experiments), or CD11b (six independent experiments). **(B)** OARSI scores were evaluated in three independent experiments and **(C)** cartilage degeneration scores were evaluated in four independent experiments. **(D, E)** IHC staining intensity was scored as absent (no staining, score 0), weak (1–20% staining, score 1), weak-moderate (21–40%, score 2), moderate (41–60%, score 3), moderate intensity (61–80%, score 4) and strong (81–100%, score 5). IHC staining data were scored in six independent experiments for both VCAM-1 and CD11b expression. ** *p* < 0.01, **** *p* < 0.0001 compared with Controls; ^##^ *p* < 0.01, ^####^ *p* < 0.0001 compared with ACLT rats.

Histopathological images showed severe synovial hypertrophy and cartilage destruction in the ACLT and ACLT + NC mimic groups, which were mitigated by miR-150-5p mimic administration (**Figure 7A**). IHC staining revealed significantly increased levels of VCAM-1 and CD11b expression in synovial tissue from the ACLT and the ACLT + NC mimic groups compared with tissue from the control group and showed that miR-150-5p significantly reduced VCAM-1 and CD11b expression (**Figure 7A**). Osteoarthritis Research Society International (OARSI) scores and the extent of cartilage degeneration were significantly higher in the ACLT and the ACLT + NC mimic groups; scores were significantly reduced by miR-150-5p mimic administration (**Figures 7B, C**). IHC staining revealed that VCAM-1 and CD11b expression was significantly increased in OA synovial tissue from the ACLT and the ACLT + NC mimic groups compared with the control group; administration of miR-150-5p mimic significantly decreased VCAM-1 and CD11b expression in OA synovial tissue (**Figures 7D, E**). These findings suggest that ACLT-induced histological changes can be reversed by miR-150-5p mimic.

## Discussion

XIST is a large transcript RNA, with approximately 17 kb localized in the X chromosome inactivation center (XIC) of chromosome Xq13.2 (45). The function of XIST is to regulate the initiation of the XIC process, which results in the heritable silencing of one of the X chromosomes during female cell development (46). Much evidence indicates that aberrant expression of XIST is associated with many human diseases, including OA (47–49). For instance, the upregulation of XIST exerts a sponge effect on miR-376c-5p by enhancing the production of cytokine OPN from M1 macrophages in OA chondrocytes (29). Moreover, a high level of XIST expression promotes apoptosis and extracellular matrix degradation of chondrocytes, while silencing of XIST impedes OA progression (28). In our bioinformatics analysis, levels of XIST were significantly elevated in human OA synovial tissue compared with healthy non-OA tissue. Knockdown of XIST expression significantly impeded VCAM-1-dependent monocyte adherence to OASFs. Our results revealed that high levels of XIST not only exist in OA chondrocytes but also in the synovial cells. Downregulation of XIST impedes VCAM-1-dependent monocyte adherence to OASFs, affecting the inflammatory microenvironment. However, whether M1 macrophages participate in XIST regulation remains to be clarified.

The distinguishing feature of miRNAs is their ability to target several genes and affect multiple biological functions (50, 51). miR-150-5p has been reported to closely interact with LINC0051, enhancing apoptosis and extracellular matrix (ECM) synthesis in OA chondrocytes (52). Our previous study demonstrated that apelin suppressed miR-150-5p expression and thereby promoted vascular endothelial growth factor (VEGF)-dependent angiogenesis (41), suggesting that miR-150-5p is an essential regulator of angiogenesis in OASFs. Of the several miRNA-seq and microarray platforms holding human OA specimens that have been examined for miRNA expression (GSE143514, GSE205684, GSE183188, GSE175961, GSE126677), no miRNA has been identified as the most likely regulator of monocyte adhesion. In this study, we analyzed the RNA-seq platform GSE143514 and used ENCORI and miRWalk2.0 miRNA prediction software to select miRNAs that potentially interact with XIST and interfere with VCAM-1 expression. The analysis revealed miR-150-5p as a prospective candidate, which was supported by the finding of lower miR-150-5p expression in OA synovial tissue compared with normal healthy synovial tissue. Upregulation of miR-150-5p significantly inhibited VCAM-1-mediated monocyte adherence to OASFs. Moreover, XIST downregulation suppressed monocyte adherence to OASFs and was reversed by the miR-150-5p inhibitor. Our evidence indicates that miR-150-5p plays an important role in controlling VCAM-1-dependent monocyte adherence to OASFs. When we used the ACLT rat model to verify the importance of miR-150-5p, we found that administration of miR-150-5p effectively alleviates ACLT-induced joint damage. It appears that low levels of miR-150-5p facilitate VEGF-mediated angiogenesis and promote monocyte adherence to OASFs, which subsequently accelerates the development of OA disease. Although miR-150-5p is yet to be validated in clinical trials, this molecule appears promising for treating OA disease.

Synovial inflammation is a critical characteristic of OA and is related to the abundance of accumulated monocytes at the inflamed site, which establishes the OA joint microenvironment (10, 11, 53). CAMs are the major modulator in synovial cells that attract monocyte infiltration and migration into inflamed synovium during OA progression (16). Cadherins, the immunoglobulin superfamily (IgSF), integrins and selectins are the four major groups of CAMs. Previous research has reported that visfatin-induced ICAM-1 production promotes monocyte adherence to human OASFs, suggesting that downregulation of adhesion molecules is a worthwhile strategy for restraining the inflammatory response and ameliorating OA symptoms (15). ICAM-1 and VCAM-1 are essential adhesion molecules that participate in leukocyte (lymphocyte/monocyte) rolling and modulate leukocyte adhesion facilitating inflammatory responses (54). ICAM-1 mainly interacts with its ligand lymphocyte function-associated antigen 1 (LFA-1), the major integrin expressed in lymphocytes (54). Similarly, VCAM-1 expression increases during inflammation, contributing to leukocyte adhesion at the inflamed site by interacting with the VCAM-1 ligand VLA-4 (55). VCAM-1-VLA-4-dependent leukocyte attraction is generally observed with monocytes and monocyte-like cell lines (56). Previous research has indicated that miR-150-5p regulates the function of lymphoid cells *via* the ICAM-1/p38/MAPK signaling pathway in allergic rhinitis (57). Until now, no studies have examined whether miR-150-5p regulates CAM-dependent leukocyte adherence in OASFs. Although the miRWalk2.0 prediction software reveals that ICAM-1 and VCAM-1 share a highly similar seed sequence for miR-150-5p, we observed much higher levels of VCAM-1 expression compared with ICAM-1 expression in OA synovial tissue, suggesting that *VCAM-1* is a potential target gene for miR-150-5p in OA patients. Certainly, upregulation of miR-150-5p significantly suppressed VCAM-1-mediated monocyte adherence to OASFs. Moreover, we found that administration of miR-150-5p effectively attenuated joint destruction in the ACLT rats and reduced VCAM-1 expression in rat synovial tissue. Whether miR-150-5p regulates lymphocyte/monocyte adhesion to OASFs by directly targeting ICAM-1 is a worthwhile study for future investigation.

**Figure 8:**
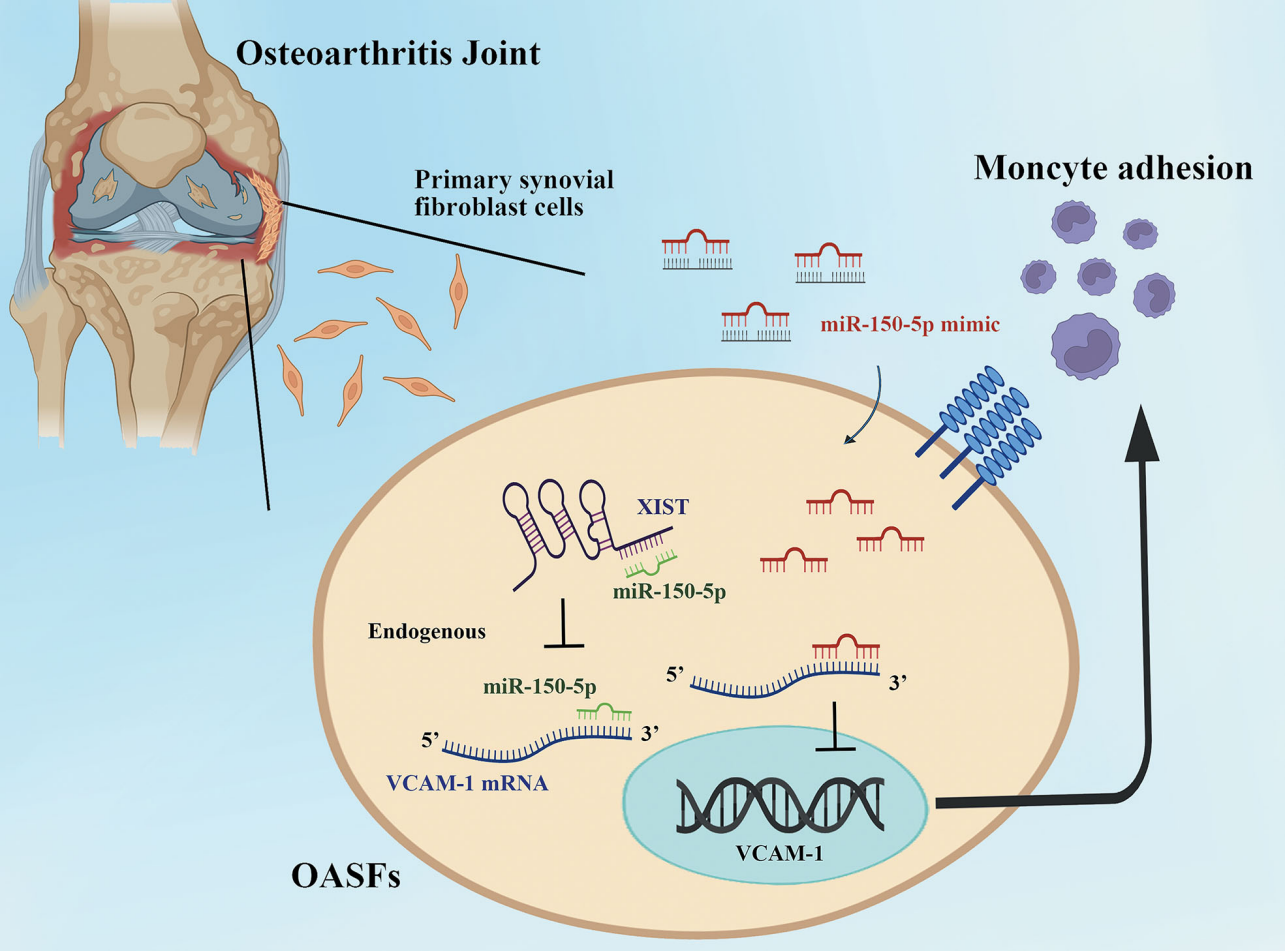
The schematic diagram summarizes the miR-150-5p and XIST interaction controls monocyte adherence in OASFs. Upregulation of miR-150-5p interferes with XIST regulation and thereby enhances VCAM-1-dependent monocyte adherence to OASFs.

In conclusion, we demonstrate that upregulation of miR-150-5p interferes with XIST regulation and thereby enhances VCAM-1-dependent monocyte adherence to OASFs (**Figure 8**). Our study is the first to demonstrate that miR-150-5p administration effectively lowers VCAM-1 expression and reduces monocyte recruitment in OASFs. Furthermore, miR-150-5p administration reduced levels of VCAM-1 and CD11b expression, which effectively attenuated OA severity in ACLT rats. Our results provide a novel insight into how XIST and miR-150-5p interact to regulate VCAM-1-dependent monocyte adherence in OA. miR-150-5p appears to be a promising biomarker for clinical diagnosis, as well as a novel miRNA therapeutic for OA treatment.

## Data availability statement

The datasets presented in this study can be found in online repositories. The names of the repository/repositories and accession number(s) can be found in the article/**Supplementary Material**.

## Ethics statement

The studies involving human participants were reviewed and approved by IRB of China Medical University Hospital. The patients/participants provided their written informed consent to participate in this study. The animal study was reviewed and approved by Institutional Animal Care and Use Committee of China Medical University.

## Author contributions

Conceptualization: Y-HW and C-HTa; Experiment and data curation: Y-HW and J-WC; Resources: C-HTs and H-TC; Funding acquisition: C-YK and C-JH; Supervision: S-CL, T-KC, and C-HTa; Writing: Y-HW. All authors agree with the published version of the manuscript.

## Funding

This study was supported by grants from Taiwan’s Ministry of Science and Technology (MOST 110-2320-B-039 -022-MY3; 110-2314-B-039-008-; 110-2314-B-195-003; 110-2314-B-039- 012), China Medical University Hospital (DMR-111-117; DMR-111-235; DMR-111-107; DMR-111-109; DMR-111-165) and China Medicine University (CMU111-MF-13).

## Acknowledgments

We thank Iona J. MacDonald from China Medical University for the editing of this paper.

## Conflict of interest

The authors declare that the research was conducted in the absence of any commercial or financial relationships that could be construed as a potential conflict of interest.

## Publisher’s note

All claims expressed in this article are solely those of the authors and do not necessarily represent those of their affiliated organizations, or those of the publisher, the editors and the reviewers. Any product that may be evaluated in this article, or claim that may be made by its manufacturer, is not guaranteed or endorsed by the publisher.

## References

[1] Ton G Yang YC Lee LW Ho WC Chen YH Yen HR. Acupuncture decreased the risk of coronary heart disease in patients with osteoarthritis in Taiwan: A nationwide matched cohort study. J Altern Complement Med (2021) 27:S60–70. doi: 10.1089/acm.2020.0153 32744906

[2] Katz JN Arant KR Loeser RF. Diagnosis and treatment of hip and knee osteoarthritis: A review. JAMA (2021) 325:568–78. doi: 10.1001/jama.2020.22171 PMC8225295 33560326

[3] Liu SC Tsai CH Wang YH Su CM Wu HC Fong YC. Melatonin abolished proinflammatory factor expression and antagonized osteoarthritis progression *in vivo* . Cell Death Dis (2022) 13:215. doi: 10.1038/s41419-022-04656-5 35256585 PMC8901806

[4] Rice SJ Beier F Young DA Loughlin J. Interplay between genetics and epigenetics in osteoarthritis. Nat Rev Rheumatol (2020) 16:268–81. doi: 10.1038/s41584-020-0407-3 32273577

[5] Ali SA Peffers MJ Ormseth MJ Jurisica I Kapoor M. The non-coding RNA interactome in joint health and disease. Nat Rev Rheumatol (2021) 17:692–705. doi: 10.1038/s41584-021-00687-y 34588660

[6] Fathollahi A Aslani S Jamshidi A Mahmoudi M. Epigenetics in osteoarthritis: Novel spotlight. J Cell Physiol (2019) 234:12309–24. doi: 10.1002/jcp.28020 PMC13484120 30659623

[7] Tavallaee G Rockel JS Lively S Kapoor M. MicroRNAs in synovial pathology associated with osteoarthritis. Front Med (Lausanne) (2020) 7:376. doi: 10.3389/fmed.2020.00376 32850892 PMC7431695

[8] Thomson A Hilkens CMU. Synovial macrophages in osteoarthritis: The key to understanding pathogenesis? Front Immunol (2021) 12:678757. doi: 10.3389/fimmu.2021.678757 34211470 PMC8239355

[9] Mathiessen A Conaghan PG. Synovitis in osteoarthritis: current understanding with therapeutic implications. Arthritis Res Ther (2017) 19:18. doi: 10.1186/s13075-017-1229-9 28148295 PMC5289060

[10] Zhang H Cai D Bai X. Macrophages regulate the progression of osteoarthritis. Osteoarthritis Cartilage (2020) 28:555–61. doi: 10.1016/j.joca.2020.01.007 31982565

[11] Haubruck P Pinto MM Moradi B Little CB Gentek R. Monocytes, macrophages, and their potential niches in synovial joints - therapeutic targets in post-traumatic osteoarthritis? Front Immunol (2021) 12:763702. doi: 10.3389/fimmu.2021.763702 34804052 PMC8600114

[12] Li Q Liu J Liu W Chu Y Zhong J Xie Y. LOX-1 regulates p. gingivalis-induced monocyte migration and adhesion to human umbilical vein endothelial cells. Front Cell Dev Biol (2020) 8:596. doi: 10.3389/fcell.2020.00596 32793587 PMC7394702

[13] Manning JE Lewis JW Marsh LJ McGettrick HM. Insights into leukocyte trafficking in inflammatory arthritis - imaging the joint. Front Cell Dev Biol (2021) 9:635102. doi: 10.3389/fcell.2021.635102 33768093 PMC7985076

[14] Pezhman L Tahrani A Chimen M. Dysregulation of leukocyte trafficking in type 2 diabetes: Mechanisms and potential therapeutic avenues. Front Cell Dev Biol (2021) 9:624184. doi: 10.3389/fcell.2021.624184 33692997 PMC7937619

[15] Law YY Lin YM Liu SC Wu MH Chung WH Tsai CH. Visfatin increases ICAM-1 expression and monocyte adhesion in human osteoarthritis synovial fibroblasts by reducing miR-320a expression. Aging (Albany NY) (2020) 12:18635–48. doi: 10.18632/aging.103889 PMC7585076 32991325

[16] Chen WC Lin CY Kuo SJ Liu SC Lu YC Chen YL. Resistin enhances VCAM-1 expression and monocyte adhesion in human osteoarthritis synovial fibroblasts by inhibiting MiR-381 expression through the PKC, p38, and JNK signaling pathways. Cells (2020) 9 :2--4. doi: 10.3390/cells9061369 PMC7349127 32492888

[17] Han D Fang Y Tan X Jiang H Gong X Wang X. The emerging role of fibroblast-like synoviocytes-mediated synovitis in osteoarthritis: An update. J Cell Mol Med (2020) 24:9518–32. doi: 10.1111/jcmm.15669 PMC7520283 32686306

[18] Liu JF Hou SM Tsai CH Huang CY Hsu CJ Tang CH. CCN4 induces vascular cell adhesion molecule-1 expression in human synovial fibroblasts and promotes monocyte adhesion. Biochim Biophys Acta (2013) 1833:966–75. doi: 10.1016/j.bbamcr.2012.12.023 23313051

[19] Yang CR Shih KS Liou JP Wu YW Hsieh IN Lee HY. Denbinobin upregulates miR-146a expression and attenuates IL-1beta-induced upregulation of ICAM-1 and VCAM-1 expressions in osteoarthritis fibroblast-like synoviocytes. J Mol Med (Berl) (2014) 92:1147–58. doi: 10.1007/s00109-014-1192-8 25052989

[20] Ghafouri-Fard S Poulet C Malaise M Abak A Mahmud Hussen B Taheriazam A. The emerging role of non-coding RNAs in osteoarthritis. Front Immunol (2021) 12:773171. doi: 10.3389/fimmu.2021.773171 34912342 PMC8666442

[21] Su Y Wu H Pavlosky A Zou LL Deng X Zhang ZX. Regulatory non-coding RNA: new instruments in the orchestration of cell death. Cell Death Dis (2016) 7:e2333. doi: 10.1038/cddis.2016.210 27512954 PMC5108314

[22] Lu C Li Z Hu S Cai Y Peng K. LncRNA PART-1 targets TGFBR2/Smad3 to regulate cell viability and apoptosis of chondrocytes *via* acting as miR-590-3p sponge in osteoarthritis. J Cell Mol Med (2019) 23:8196–205. doi: 10.1111/jcmm.14690 PMC6850963 31571401

[23] Huang Z Zhang N Ma W Dai X Liu J. MiR-337-3p promotes chondrocytes proliferation and inhibits apoptosis by regulating PTEN/AKT axis in osteoarthritis. BioMed Pharmacother (2017) 95:1194–200. doi: 10.1016/j.biopha.2017.09.016 28931211

[24] Lopez-Urrutia E Bustamante Montes LP Ladron de Guevara Cervantes D Perez-Plasencia C Campos-Parra AD. Crosstalk between long non-coding RNAs, micro-RNAs and mRNAs: Deciphering molecular mechanisms of master regulators in cancer. Front Oncol (2019) 9:669. doi: 10.3389/fonc.2019.00669 31404273 PMC6670781

[25] Lu G Li L Wang B Kuang L. LINC00623/miR-101/HRAS axis modulates IL-1beta-mediated ECM degradation, apoptosis and senescence of osteoarthritis chondrocytes. Aging (Albany NY) (2020) 12:3218–37. doi: 10.18632/aging.102801 PMC7066905 32062610

[26] Zhong L Simard MJ Huot J. Endothelial microRNAs regulating the NF-kappaB pathway and cell adhesion molecules during inflammation. FASEB J (2018) 32:4070–84. doi: 10.1096/fj.201701536R 29565737

[27] Xie F Liu YL Chen XY Li Q Zhong J Dai BY. Role of MicroRNA, LncRNA, and exosomes in the progression of osteoarthritis: A review of recent literature. Orthop Surg (2020) 12:708–16. doi: 10.1111/os.12690 PMC7307224 32436304

[28] Liu Y Liu K Tang C Shi Z Jing K Zheng J. Long non-coding RNA XIST contributes to osteoarthritis progression *via* miR-149-5p/DNMT3A axis. BioMed Pharmacother (2020) 128:110349. doi: 10.1016/j.biopha.2020.110349 32521454

[29] Li L Lv G Wang B Kuang L. XIST/miR-376c-5p/OPN axis modulates the influence of proinflammatory M1 macrophages on osteoarthritis chondrocyte apoptosis. J Cell Physiol (2020) 235:281–93. doi: 10.1002/jcp.28968 31215024

[30] Su C-H Lin C-Y Tsai C-H Lee H-P Lo L-C Huang W-C. Betulin suppresses TNF-α and IL-1β production in osteoarthritis synovial fibroblasts by inhibiting the MEK/ERK/NF-κB pathway. J Funct Foods (2021) 86 :4. doi: 10.1016/j.jff.2021.104729

[31] Achudhan D Liu SC Lin YY Lee HP Wang SW Huang WC. Antcin K inhibits VEGF-dependent angiogenesis in human rheumatoid arthritis synovial fibroblasts. J Food Biochem (2022) 46:e14022. doi: 10.1111/jfbc.14022 34841538

[32] Lee H-P Chen P-C Wang S-W Fong Y-C Tsai C-H Tsai F-J. Plumbagin suppresses endothelial progenitor cell-related angiogenesis *in vitro* and *in vivo* . J Funct Foods (2019) 52:537–44. doi: 10.1016/j.jff.2018.11.040

[33] Lee H-P Wu Y-C Chen B-C Liu S-C Li T-M Huang W-C. Soya-cerebroside reduces interleukin production in human rheumatoid arthritis synovial fibroblasts by inhibiting the ERK, NF-κB and AP-1 signalling pathways. Food Agric Immunol (2020) 31:740–50. doi: 10.1080/09540105.2020.1766426

[34] Chen WC Lu YC Kuo SJ Lin CY Tsai CH Liu SC. Resistin enhances IL-1beta and TNF-alpha expression in human osteoarthritis synovial fibroblasts by inhibiting miR-149 expression *via* the MEK and ERK pathways. FASEB J (2020) 34:13671–84. doi: 10.1096/fj.202001071R 32790946

[35] Lee H-P Liu S-C Wang Y-H Chen B-C Chen H-T Li T-M. Cordycerebroside a suppresses VCAM-dependent monocyte adhesion in osteoarthritis synovial fibroblasts by inhibiting MEK/ERK/AP-1 signaling. J Funct Foods (2021) 86 :2. doi: 10.1016/j.jff.2021.104712

[36] Liu S-C Tsai C-H Wu T-Y Tsai C-H Tsai F-J Chung J-G. Soya-cerebroside reduces IL-1β-induced MMP-1 production in chondrocytes and inhibits cartilage degradation: implications for the treatment of osteoarthritis. Food Agric Immunol (2019) 30:620–32. doi: 10.1080/09540105.2019.1611745

[37] Chang AC Chen PC Lin YF Su CM Liu JF Lin TH. Osteoblast-secreted WISP-1 promotes adherence of prostate cancer cells to bone *via* the VCAM-1/integrin alpha4beta1 system. Cancer Lett (2018) 426:47–56. doi: 10.1016/j.canlet.2018.03.050 29627497

[38] Choi J Lee HE Kim MA Jang BG Lee HS Kim WH. Analysis of MET mRNA expression in gastric cancers using RNA *in situ* hybridization assay: its clinical implication and comparison with immunohistochemistry and silver *in situ* hybridization. PLoS One (2014) 9:e111658. doi: 10.1371/journal.pone.0111658 25364819 PMC4218795

[39] Lee KT Su CH Liu SC Chen BC Chang JW Tsai CH. Cordycerebroside a inhibits ICAM-1-dependent M1 monocyte adhesion to osteoarthritis synovial fibroblasts. J Food Biochem (2022) 46:e14108. doi: 10.1111/jfbc.14108 35165902

[40] Liu SC Chiu CP Tsai CH Hung CY Li TM Wu YC. Soya-cerebroside, an extract of cordyceps militaris, suppresses monocyte migration and prevents cartilage degradation in inflammatory animal models. Sci Rep (2017) 7:43205. doi: 10.1038/srep43205 28225075 PMC5320555

[41] Wang YH Kuo SJ Liu SC Wang SW Tsai CH Fong YC. Apelin affects the progression of osteoarthritis by regulating VEGF-dependent angiogenesis and miR-150-5p expression in human synovial fibroblasts. Cells (2020) 9 :5. doi: 10.3390/cells9030594 PMC7140420 32131466

[42] Wang CJ Cheng JH Chou WY Hsu SL Chen JH Huang CY. Changes of articular cartilage and subchondral bone after extracorporeal shockwave therapy in osteoarthritis of the knee. Int J Med Sci (2017) 14:213–23. doi: 10.7150/ijms.17469 PMC5370283 28367081

[43] Chen CY Su CM Hsu CJ Huang CC Wang SW Liu SC. CCN1 promotes VEGF production in osteoblasts and induces endothelial progenitor cell angiogenesis by inhibiting miR-126 expression in rheumatoid arthritis. J Bone Miner Res (2017) 32:34–45. doi: 10.1002/jbmr.2926 27465842

[44] Fernandes JCR Acuna SM Aoki JI Floeter-Winter LM Muxel SM. Long non-coding RNAs in the regulation of gene expression: Physiology and disease. Noncoding RNA (2019) 5(1):17. doi: 10.3390/ncrna5010017 PMC6468922 30781588

[45] Brockdorff N. Localized accumulation of xist RNA in X chromosome inactivation. Open Biol (2019) 9:190213. doi: 10.1098/rsob.190213 31795917 PMC6936258

[46] Wang W Min L Qiu X Wu X Liu C Ma J. Biological function of long non-coding RNA (LncRNA) xist. Front Cell Dev Biol (2021) 9:645647. doi: 10.3389/fcell.2021.645647 34178980 PMC8222981

[47] Wang Y Liang Y Luo J Nie J Yin H Chen Q. XIST/miR-139 axis regulates bleomycin (BLM)-induced extracellular matrix (ECM) and pulmonary fibrosis through beta-catenin. Oncotarget (2017) 8:65359–69. doi: 10.18632/oncotarget.18310 PMC5630336 29029436

[48] Xiao L Gu Y Sun Y Chen J Wang X Zhang Y. The long noncoding RNA XIST regulates cardiac hypertrophy by targeting miR-101. J Cell Physiol (2019) 234:13680–92. doi: 10.1002/jcp.28047 PMC13483095 30605239

[49] Ma M Pei Y Wang X Feng J Zhang Y Gao MQ. LncRNA XIST mediates bovine mammary epithelial cell inflammatory response *via* NF-kappaB/NLRP3 inflammasome pathway. Cell Prolif (2019) 52:e12525. doi: 10.1111/cpr.12525 30362186 PMC6430464

[50] Stavast CJ Erkeland SJ. The non-canonical aspects of MicroRNAs: Many roads to gene regulation. Cells (2019) 8 :9-13. doi: 10.3390/cells8111465 PMC6912820 31752361

[51] Kabekkodu SP Shukla V Varghese VK Chakrabarty S Satyamoorthy K. Clustered miRNAs and their role in biological functions and diseases. Biol Rev Camb Philos Soc (2018) 9–13:1955–86. doi: 10.1111/brv.12428 29797774

[52] Zhang Y Dong Q Sun X. Positive feedback loop LINC00511/miR-150-5p/SP1 modulates chondrocyte apoptosis and proliferation in osteoarthritis. DNA Cell Biol (2020) 39:1506–12. doi: 10.1089/dna.2020.5718 32635763

[53] Khamchun S Thakaeng C Na Lampang R. Serum alpha-1-antitrypsin level in the severity prognosis of systemic lupus erythematosus patients: Systematic exploration of novel biomarker. Biomedicine (Taipei) (2022) 12:19–30. doi: 10.37796/2211-8039.1297 35836976 PMC9236717

[54] Harjunpaa H Llort Asens M Guenther C Fagerholm SC. Cell adhesion molecules and their roles and regulation in the immune and tumor microenvironment. Front Immunol (2019) 10:1078. doi: 10.3389/fimmu.2019.01078 31231358 PMC6558418

[55] Kourtzelis I Mitroulis I von Renesse J Hajishengallis G Chavakis T. From leukocyte recruitment to resolution of inflammation: the cardinal role of integrins. J Leukoc Biol (2017) 102:677–83. doi: 10.1189/jlb.3MR0117-024R PMC5557641 28292945

[56] Ley K Laudanna C Cybulsky MI Nourshargh S. Getting to the site of inflammation: the leukocyte adhesion cascade updated. Nat Rev Immunol (2007) 7:678–89. doi: 10.1038/nri2156 17717539

[57] Zhang L Meng W Chen X Ning Y Sun M Wang R. MiR-150-5p regulates the functions of type 2 innate lymphoid cells *via* the ICAM-1/p38 MAPK axis in allergic rhinitis. Mol Cell Biochem (2022) 477:1009–22. doi: 10.1007/s11010-021-04346-4 34988856

