# Supplementary material for: miR-150-5p and XIST interaction controls monocyte adherence: Implications for osteoarthritis therapy
Source: Front Immunol. 2022 Sep 20;13:1004334. doi: 10.3389/fimmu.2022.1004334 (PMC9530358; doi:10.3389/fimmu.2022.1004334)
Supplement: Supplementary file 1 [file DataSheet_1.docx]

Supplementary Material

**Materials**

Antibodies against CD11b and β-actin were purchased from GeneTex (Hsinchu, Taiwan). VCAM-1 antibody was obtained from Sigma-Aldrich (St. Louis, MO, USA). Small interfering RNAs (siRNAs) against XIST (si-XIST) were purchased from TrustGene Bio Inc. (Taipei, Taiwan). Negative control siRNA (si-NC) and VCAM-1 siRNA (si-VCAM-1) were purchased from Santa Cruz Biotechnology, Inc. (Dallas, TX, USA). All the miRNA mimics, inhibitors and their corresponding controls (negative control mimic; NC mimic [ the miRNA negative control has no homology to any known miRNA or mRNA sequences] and negative control inhibitor; NC inhibitor) were obtained from AllBio Science Inc. (Taipei, Taiwan). Plasmid constructs were commercially synthesized by MDBio, Inc. (Taipei, Taiwan). The VCAM-1 overexpression plasmid (VCAM-1 OV) was commercially synthesized by NCFB, Academia Sinica (Taipei, Taiwan).

**Clinical synovium and primary cell culture**

OA synovial tissues were obtained from 20 patients (8 males and 12 females) receiving knee joint arthroplasty, and normal synovial tissues were obtained from 7 OA patients who underwent arthroscopy and were diagnosed with joint trauma (sports injury or an accident). The protocol for this study was approved by the Institutional Review Board of China Medical University Hospital. The informed consent forms were signed by all patients before they participated in this research. OASFs were digested in the collagenase solution and cultured in DMEM medium, as previously described. Human fibroblast-like synoviocytes (HFLSs) isolated from normal synovial tissues were purchased from Cell Applications, Inc. (San Diego, CA, USA). HEK-293 cells were purchased from ATCC (Manassas, VA, USA).

THP-1 monocytic cells were obtained from the American Type Culture Collection (Manassas, VA, USA) and cultured in RPMI-1640 medium (Gibco, USA) containing 4.5 g/L glucose, 10 mM HEPES, 1 mM sodium pyruvate, 1.5 g/L sodium bicarbonate and 10% fetal bovine serum (FBS) supplemented with 0.05 mM 2-mercaptoethanol.

**Total RNA isolation and quantitative reverse transcription PCR (RT-qPCR)**

Total RNA was extracted from normal, OA synovial tissues and OASFs by TRIzol reagent (MDBio Inc., Taipei, Taiwan). Reverse transcription of total RNA 2μg into complementary DNA (cDNA) was performed with the M-MLV RT kit (Thermo Fisher Scientific; Waltham, MA, USA) and the Mir-X™ miRNA First-Strand Synthesis kit (Terra Bella Avenue; Mountain View, CA, USA), following the manufacturers’ instructions. Sequence-specific primers for all target gene primers were purchased commercially. RT-qPCR assays were conducted using the StepOnePlus™ Real-Time PCR System (Applied Biosystems).

Primer sequences were as follows:

human VCAM-1 (forward: 5'-TTCCAGGGACTTCCTGTCTG-3' and reverse: 5'-TCCGTCTCATTGACTTGCAG-3')

human GAPDH (forward: 5'-AAT GGACAACTGGTCGTGGAC-3' and reverse: 5'-CCCTCCAGGGGATCTGTTTG-3')

human XIST (forward: 5'-AGCTCCTCGGACAGCTGTAA-3' and reverse: 5'-CTCCAGATAGCTGGCAACC-3')

has-miR-150-5p (forward: 5'-TCTCCCAACCCTTGTACCAGTG-3')

U6 (forward: 5'-CGCTTCGGCAGCACATATAC-3' and reverse: 5'-AAAATATGGAACGCTTCACGA-3').

**Western blot analysis**

Cells were lysed with 100 μl RIPA lysis buffer containing a protease inhibitor cocktail (Roche, Indianapolis, IN, USA). The collection of supernatants was performed in a Western blot assay. The detailed procedures are described in our previous studies.

**Transfection and luciferase reporter assays**

OASFs were transfected for 24 h with a miRNA mimic, a miR-150-5p inhibitor, or the corresponding siRNAs using Lipofectamine 2000 (Invitrogen, Waltham, MA, USA), according to the user manual.

The luciferase plasmids have 3' untranslated region (3’UTR) of VCAM-1 or fragment sequence of XIST and both plasmids contain wild-type (WT) or mutant (MUT) miR-150-5p binding site. The luciferase plasmids were respectively co-transfected with miR-150-5p mimic or NC mimic in OASFs for 24 h. Luciferase activity was monitored using the dual luciferase assay system, following the manufacturer’s instructions. Luciferase activity was assessed by a luminometer, using the method described in our previous publication.

**Immunohistochemistry (IHC) staining**

IHC staining was performed on serial sections of the knee joints of the rats and human synovial tissues. The sections were deparaffinized with xylene and rehydrated with ethanol, then stained with specific primary antibodies (1:200) overnight at 4°C, according to our previous protocol. The specimens were also stained with hematoxylin and eosin (H&E) and Safranin O-fast Green for histopathological changes. The OARSI scoring system followed Carlson.^1^ IHC staining intensity was scored by two independent observers blinded to histopathologic data. The staining intensities of the samples were divided into five categories: absent (no staining, score 0), weak (1–20% staining, score 1), weak-moderate (21–40%, score 2), moderate (41–60%, score 3), moderate-strong (61–80%, score 4), and strong (81–100%, score 5).

**References**

1. Gerwin, N, Bendele, AM, Glasson, S, and Carlson, CS (2010). The OARSI histopathology initiative - recommendations for histological assessments of osteoarthritis in the rat. *Osteoarthritis Cartilage* **18 Suppl 3**: S24-34.
